# Artesunate may inhibit liver fibrosis via the FAK/Akt/β-catenin pathway in LX-2 cells

**DOI:** 10.1186/s40360-018-0255-9

**Published:** 2018-10-16

**Authors:** Jian Lv, Ruidan Bai, Li Wang, Jiefang Gao, Hong Zhang

**Affiliations:** Department of Pharmacy, Renmin Hospital of Wuhan University, Zhang Zhidong Road, Wuhan, Hubei 430060 People’s Republic of China

**Keywords:** Artesunate, Liver fibrosis, Hepatic stellate cells, Activation, FAK/Akt/β-catenin

## Abstract

**Background:**

An increasing number of studies are investigating the effects of Chinese medicine on hepatic fibrosis, but only few studies have examined the anti-fibrogenic properties of Artesunate (ART). The aim of the present study was to explore the anti-fibrotic effects of ART on LX-2 cells, the human HSC cell line, and to determine potential molecular mechanisms via the focal adhesion kinase (FAK)/ protein kinase B (Akt)/ β-catenin pathway.

**Methods:**

LX-2 cells were stimulated with different concentration of ART (0, 12.5, 25 and 50 μg/ml) for 12, 24, 48 or 72 h, their proliferation was analyzed using the Cell Counting Kit-8 (CCK-8) assay. LX-2 cells were treated with different doses of ART (0, 12.5, 25 and 50 μg/ml) for 24 h, their apoptosis was measured using flow cytometry, the levels of mRNAs encoding collagen I or α-smooth muscle actin (α-SMA) were determined using reverse transcription-quantitative polymerase chain reaction (RT-qPCR) and the levels of key proteins in the FAK/Akt/β-catenin signaling pathway were assessed by western blotting. Specific inhibitors of FAK were added to the LX-2 cells cultures to explore the potential signaling.

**Results:**

Exposing LX-2 cells to ART efficiently inhibited their proliferation, significantly promoted early apoptosis in a dose-dependent manner, and markedly downregulated the mRNA expression of α-SMA and collagen I. In addition, ART, similar to FAK inhibitor PF562271 significantly inhibited the FAK/Akt/β-catenin signaling pathway by reducing the levels of phosphorylated FAK, Akt and GSK-3β.

**Conclusions:**

Our present study shows that ART could regulate the proliferation, apoptosis and activation of LX-2. Meanwhile, the anti-fibrogenic mechanisms of ART was correlated with FAK/Akt/β-catenin pathway. Future research should verify and extend these findings, as well as explore other molecules and therefore serve as useful therapeutic targets.

## Background

Hepatic fibrosis is a compensatory response to chronic liver injury and inflammation arising from various chronic insults, which may progress into liver cirrhosis and even liver cancer if the underlying causes persist [1–3]. The onset and progression of hepatic fibrosis are driven by hepatic stellate cells (HSCs), which are stimulated by cytokines and reactive oxygen species to produce excessive extracellular matrix (ECM) [4–6].

One of the multiple signaling pathways that drive liver fibrosis progression is the focal adhesion kinase (FAK) signaling pathway. In fact, FAK has been shown to help regulate the prolifetation [7, 8], activation [9, 10] and apoptosis [11, 12] of HSCs. In addition, several studies suggested that inhibition of FAK/Akt signaling by drugs [13, 14] or microRNAs [15] may be a potential target for the prevention of liver fibrosis. Furthermore, upregulation in Wnt/β-catenin signaling may lead to activation of HSC [16], whereas inhibition of Wnt/β-catenin signaling may arrest the progression of liver fibrosis using small interfering RNA [17]. Under normal physiological conditions, β-catenin combines with GSK-3β to form cell-cell adhesion complexes that transmit a contact inhibition signal, thereby maintaining HSCs in a quiescent state [16, 18]. Taken together, the findings of several studies point to a pathway in which drug-mediated downregulation of the Wnt/β-catenin signaling pathway inhibits HSC activation in vitro and in vivo [19–21].

Artesunate (ART), a small molecule derived from Artemisinin extracted from the traditional Chinese herb *Artemisia annua,* markedly inhibits the progression of hepatic fibrosis by downregulating matrix metalloproteinases 2 and 9 in rats induced by bovine serum albumin [22]. However, only few published studies have investigated the anti-fibrogenic properties of ART [23, 24]. In myelodysplastic syndrome (MDS) cells (SKM-1 cells), ART regulates apoptosis by inhibiting the expression of downstream targets of Wnt/β-catenin signaling pathway, such as c-Myc and cyclin D1 [25]. We have demonstrated that, in HSC-T6 cells, ART inhibited the phosphorylation of GSK-3β and downregulated β-catenin [26], whereas more recently we found that Akt acts upstream of the Wnt/β-catenin pathway [27], suggesting that ART may exert its anti-fibrogenic effects primarily through the Akt/β-catenin pathway, which was consistent with the findings of Kamo et al [28]. In summary, it is important to determine whether the anti-fibrotic effect of ART is associated with FAK, and whether this inhibition occurs via the FAK/Akt/β-catenin pathway.

In order to verify and extend these results in a different cellular context, we investigated the effect of ART on the proliferation, apoptosis and activation of the human HSC cell line LX-2. We specifically examined whether FAK acts upstream of the Akt/β-catenin pathway and whether ART regulates the FAK/Akt/β-catenin pathway to exert its anti-fibrogenic effects. The results of the present study may provide new insight into the treatment of liver fibrosis.

## Methods

### Reagents

ART was purchased from Guilin Pharmaceutical (Shanghai, China), dissolved in dimethyl sulfoxide (DMSO) and stored at − 80 °C. The concentration of DMSO in all experiments was 0.1%. The FAK inhibitor PF562271 was purchased from Selleck Chemicals (Houston, TX, USA). CCK-8 was obtained from BioSharp (Wuhan, China). The PE-Annexin V/7-AAD kit was obtained from BD Biosciences (Franklin Lakes, NJ, USA). TRIzol reagent was purchased form Invitrogen/Thermo Fisher Scientific (Carlsbad, CA, USA). Prime Script RT reagent Kit and SYBR® Premix Ex Taq™ were purchased from Takara (Tokyo, Japan). The BCA protein assay kit was from Pierce/Thermo Fisher Scientific (Waltham, MA, USA). Primary antibodies against FAK (# 3285), p-FAK (# 3283), Akt (# 4691), p-Akt (# 4060), GSK-3β (# 9315), p-GSK-3β (# 9336), β-catenin (# 9562), Bax (# 2772), Bcl-2 (# 3498) and GAPDH (# 2118) were obtained from Cell Signaling Technology Inc. (Danvers, MA, USA). Anti-rabbit secondary antibody IgG H + L (# 5151) was purchased from Cell Signaling Technology Inc.

### Cell culture

The human HSC cell line LX-2 was purchased from Beijing North Carolina Souren Biotechnology (BNCC337957; Beijing, China). Cells were cultured in DMEM/high glucose medium (HyClone/GE Healthcare, Logan, UT, USA) supplemented with 10% fetal bovine serum (Gibco/Thermo Fisher Scientific), 100 U/ml penicillin and 100 μg/ml streptomycin at 37 °C in a humidified incubator with 5% CO_2._

### Cell viability assay

Briefly, the cells were seeded into 96-well plates (5 × 10^3^ cells/well) and incubated overnight. The medium was replaced with fresh medium containing different concentrations of ART (0, 12.5, 25 and 50 μg/ml) or FAK inhibitor (0, 1, 3, 5 and 7 μmol/l). After 12, 24, 48 or 72 h, CCK-8 reagent (10 μl) was added to each well and the plates were cultured at 37 °C for another 30 min. Absorbance at 450 nm [optical density (OD)_450_] was measured using a microplate reader (MR7000, Dynatech, Edgewood, NY, USA). Growth inhibition was calculated as follows: CV = (OD_450,test_ /OD_450,control_) × 100%.

### Flow cytometry

Cells were seeded into 6-well plates (2 × 10^5^ cells/well) and incubated with different concentrations of ART (0, 12.5, 25 and 50 μg/ml) at 37 °C for 24 h. Floating and adherent cells were collected, washed twice with cold phosphate-buffered saline (PBS), and assessed for apoptosis using the PE-Annexin V/7-AAD kit, followed by flow cytometry (BD Biosciences, San Jose, CA, USA). Cells staining positive for PE-annexin V and negative for 7-AAD were considered to be in early apoptosis, while those positive for both PE-annexin V and 7-AAD were considered as late apoptotic cells.

### RT-qPCR

Cells were seeded into 6-well plates (2 × 10^5^ cells/well) and stimulated with different concentrations of ART (0, 12.5, 25 and 50 μg/ml) for 24 h, then harvested. Total RNA was isolated using TRIzol reagent, and cDNA was synthesized using the Prime Script RT reagent kit. RT-qPCR was performed using SYBR® Premix Ex Taq™ and a real-time PCR system (Applied Biosystems 7500; Applied Biosystems/Thermo Fisher Scientific). The thermocycling conditions were denaturation at 95 °C for 5 s, followed by annealing at 60 °C for 40 s, for a total of 40 cycles. The condition of the melting curve were 95 °C for 40 s, then decreased to 60 °C in 1 min. The levels of mRNAs encoding α-SMA or collagen I were normalized to the levels of GAPDH mRNA, and relative expression was calculated according to the 2^-∆∆Cq^ method. The primer sequences were as follows: α-SMA, forward 5’-ACGAGACCACCTACAACAGCAT-3′ and reverse 5’-CTCGTCGTACTCCTGCTTGGT-3′; collagen I, forward 5’-ACTGGTGAGACCTGCGTGTA-3′ and reverse 5’-AATCCATCGGTCATGCTCTC-3′; GAPDH, forward 5’-ATGACATCAAGAAGGTGGTG-3′ and reverse 5’-CATACCAGGAAATGAGCTTG-3′.

### Western blotting

Cells were seeded into 6-well plates (2 × 10^5^ cells/well) and incubated with different concentrations of ART (0, 12.5, 25 and 50 μg/ml) for 24 h, then harvested. The cells were treated with 3 μM PF562271 alone, 25 μg/ml ART alone, or the combination of 25 μg/ml ART and 3 μM PF562271 for 24 h, then harvested. The collected LX-2 cells were lysed for 30 min at 4 °C in RIPA buffer containing protease inhibitor cocktail, phenylmethanesulfonyl fluoride, and phosphorylated protease inhibitors A and B. Total protein concentration was quantified using the BCA protein assay kit. Proteins were separated on 8–15% SDS-PAGE and transferred onto PVDF membranes, which were blocked with 5% bovine serum albumin for 2 h, and washed three times with Tris-buffered saline containing Tween-20 (TBST). The membranes were then incubated overnight at 4 °C with primary antibodies against FAK (ratio: 1:1,000), p-FAK (ratio: 1:1,000), Akt (ratio: 1:1,000), p-Akt (ratio: 1:1,000), GSK-3β (ratio: 1:1,000), p-GSK-3β (ratio: 1:1,000), β-catenin (ratio: 1:1,000), Bax (ratio: 1:1,000), Bcl-2 (ratio: 1:1,000), or GAPDH (ratio: 1:1,000). After three washes in TBST, the membranes were incubated for 2 h with anti-rabbit secondary antibody IgG H + L (ratio: 1:10,000). The levels of target proteins were quantified using the Odyssey system (LI-COR Biosciences, Lincoln, NE, USA) according to the manufacturer’s instructions and normalized to the levels of GAPDH.

### Statistical analyses

All experiments were repeated at least three times, and the results are presented as mean ± standard error of mean (SEM). The levels of mRNAs or protein of target gene were normalized to the levels of GAPDH mRNA or protein, respectively. The results were presented as percent increase or decrese of the control group. The results were analyzed using Student’s t-test and one-way analysis of variance using SPSS 17.0 software (SPSS Inc., Chicago, IL, USA). *P* < 0.05 was considered to indicate statistically significant differences.

## Results

### ART inhibits LX-2 cell proliferation

**Fig. 1:**
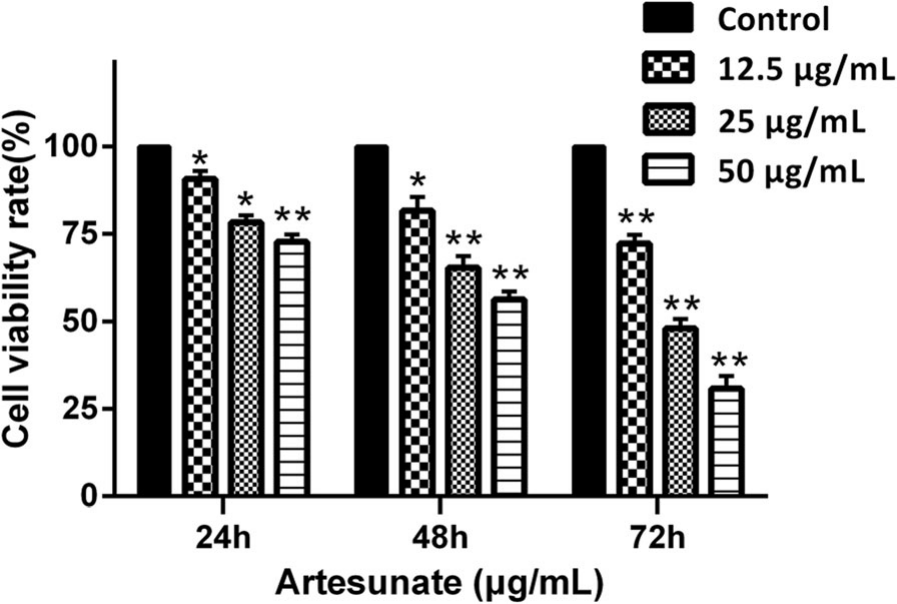
Effects of ART on LX-2 cell viability. Relative cell viability of LX-2 cells treated with ART (0, 12.5, 25 and 50 μg/ml) for 24, 48 or 72 h (*n* = 6). Viability was measured using the CCK-8 assay. Each column shows the mean ± SEM. ^*^ means *P* < 0.05 vs control. ^**^ means *P* < 0.01 vs control

LX-2 cells were treated with different concentrations of ART (0, 12.5, 25 and 50 μg/ml). After 12, 24, 48 or 72 h, proliferation was evaluated using the CCK-8 assay (Fig. 1). ART inhibited cell proliferation in a time- and dose-dependent manner, and the inhibition was significant by 24 h (P < 0.05). The IC_50_ of ART was 69.20 μg/ml at 72 h.

### Effect of ART on the apoptosis of LX-2 cells

**Fig. 2:**
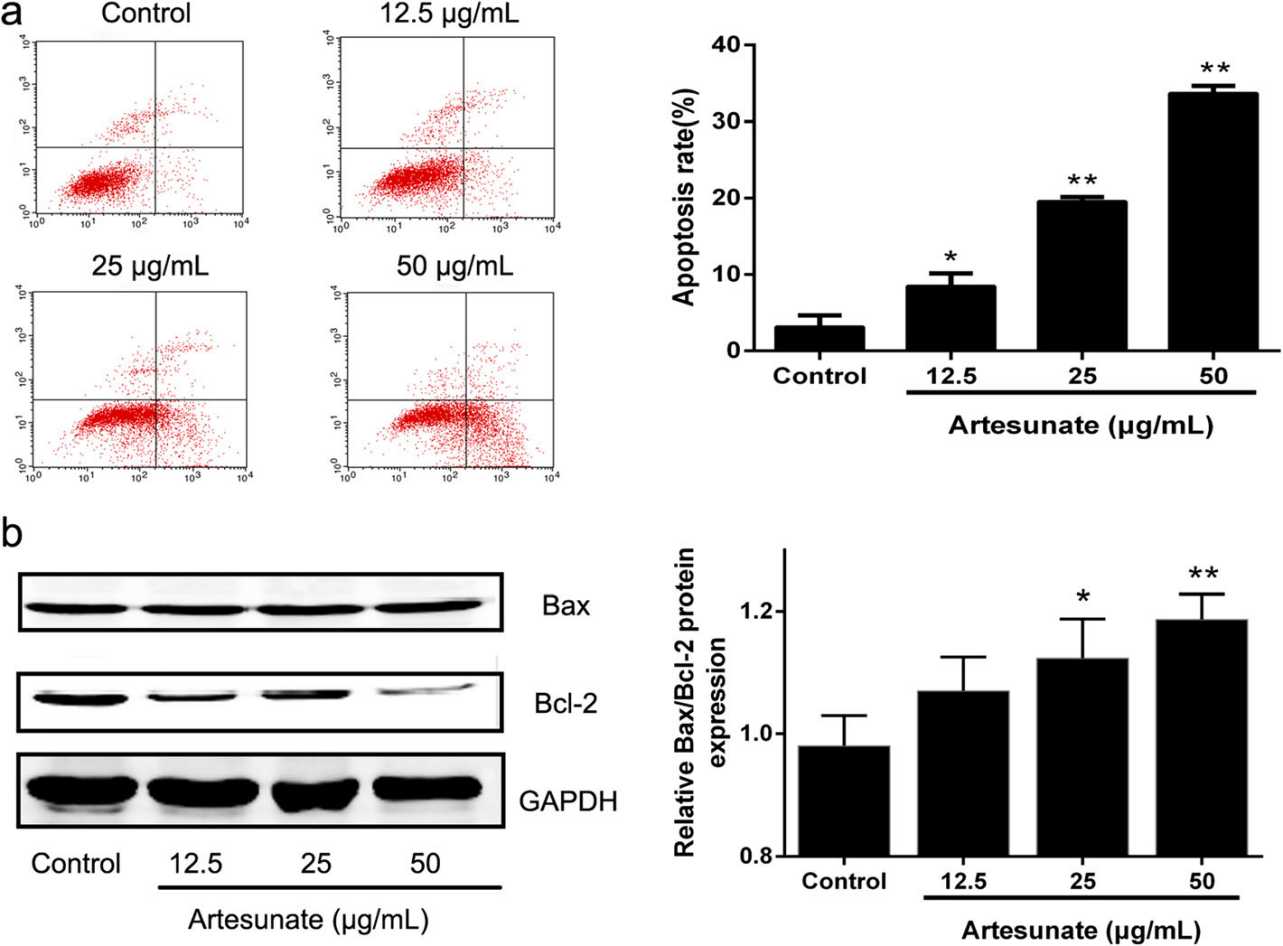
Effects of ART on LX-2 cell apoptosis. **a** Rates of cell apoptosis following 24 h exposure to ART (0, 12.5, 25 and 50 μg/ml), as analyzed using flow cytometry (*n* = 3); **b** Levels of Bax and Bcl-2 in cells treated with ART (0, 12.5, 25 and 50 μg/ml) for 24 h, analyzed using western blotting (*n* = 3). Each column shows the mean ± SEM. ^*^ means *P* < 0.05 vs control. ^**^ means *P* < 0.01 vs control

After stimulated with different concentrations of ART, the early apoptosis rate of each group were 4.54% at 12.5 μg/ml, 15.58% at 25 μg/ml and 31.72% at 50 μg/ml. ART increased significantly the apoptosis of LX-2 cells compared with the control group (Fig. 2a). In addition, ART also reduced Bcl-2 expression in a dose-dependent manner and increased the Bax/Bcl-2 ratio, which contributed to apoptosis (Fig. 2b).

### Effects of ART on activation of LX-2 cells

**Fig. 3:**
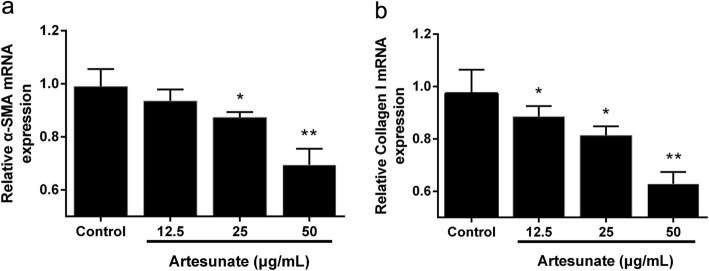
Effects of ART on activation of LX-2 cells. **a** the mRNA level of α-SMA; **b** the mRNA level of collagen I were determined by RT-qPCR (n = 3) following treatment with ART (0, 12.5, 25 and 50 μg/ml) for 24 h. Each column shows the mean ± SEM. ^*^ means *P* < 0.05 vs control. ^**^ means *P* < 0.01 vs control

The typical characteristics of HSCs activation is the excessive deposition of ECM proteins, including ɑ-SMA and collagen I. ART treatment downregulated mRNAs encoding ɑ-SMA and collagen I (Fig. 3). Our results suggest that ART exerts anti-activation effects on LX-2 cells.

### ART inhibits phosphorylation of FAK and Akt and inhibits Wnt/β-catenin signaling

**Fig. 4:**
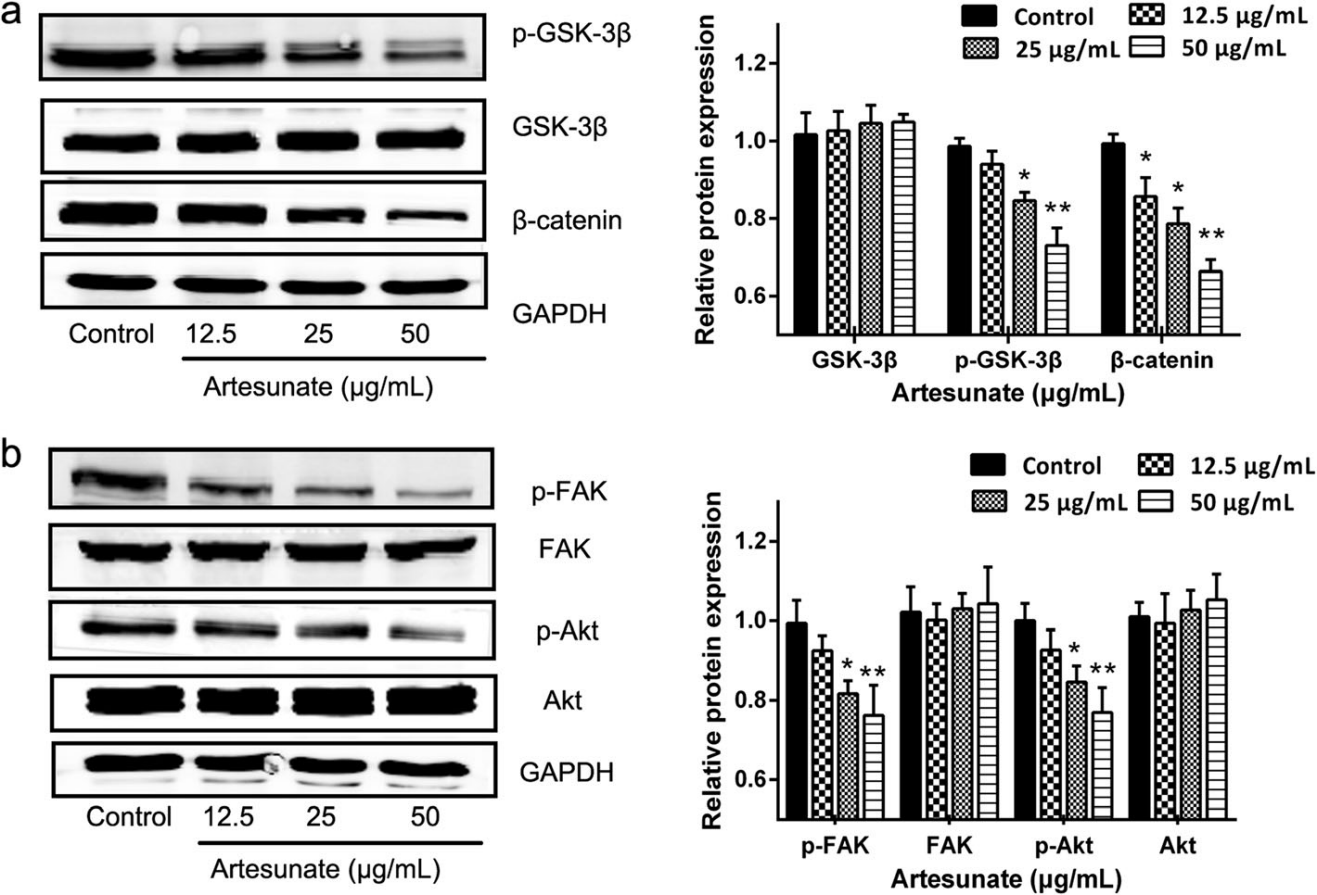
Effects of ART on the levels of FAK, p-FAK, Akt, p-Akt, GSK-3β, p-GSK-3β and β-catenin. **a** The levels of GSK-3β, phospho-GSK-3β and β-catenin were measured in cells exposed to ART (0, 12.5, 25 and 50 μg/ml) for 24 h by western blotting (*n* = 3); **b** The levels of FAK, phospho-FAK, Akt and phospho-Akt were measured in cells exposed to ART (0, 12.5, 25 and 50 μg/ml) for 24 h by western blotting (n = 3). GAPDH served as an internal control. Data are shown as mean ± SEM. ^*^ means *P* < 0.05 vs control. ^**^ means *P* < 0.01 vs control

To explore in molecular detail the mechanisms underlying the observed effects of ART on HSCs proliferation, apoptosis and activation, LX-2 cells were treated with different concentrations of ART at 24 h. Western blotting was used to assess the levels of FAK, p-FAK, Akt, p-Akt, GSK-3β, p-GSK-3β and β-catenin. ART downregulated p-GSK-3β and β-catenin to levels significantly below those in control cultures, except GSK-3β at 24 h (Fig. 4a). These results suggest that the classical Wnt/β-catenin signaling pathway is involved in the mediation of the anti-fibrogenic effects of ART. Furthermore, ART downregulated the levels of p-Akt in a concentration-dependent manner (Fig. 4b), Akt is correlated with the effects of ART, which is in accordance with the findings of previous studies. In addition, ART reduced the level of p-FAK, indicating that the anti-fibrogenic effects of ART on LX-2 cells are closely correlated with FAK gene expression.

### Effects of ART require the FAK/Akt/β-catenin pathway

**Fig. 5:**
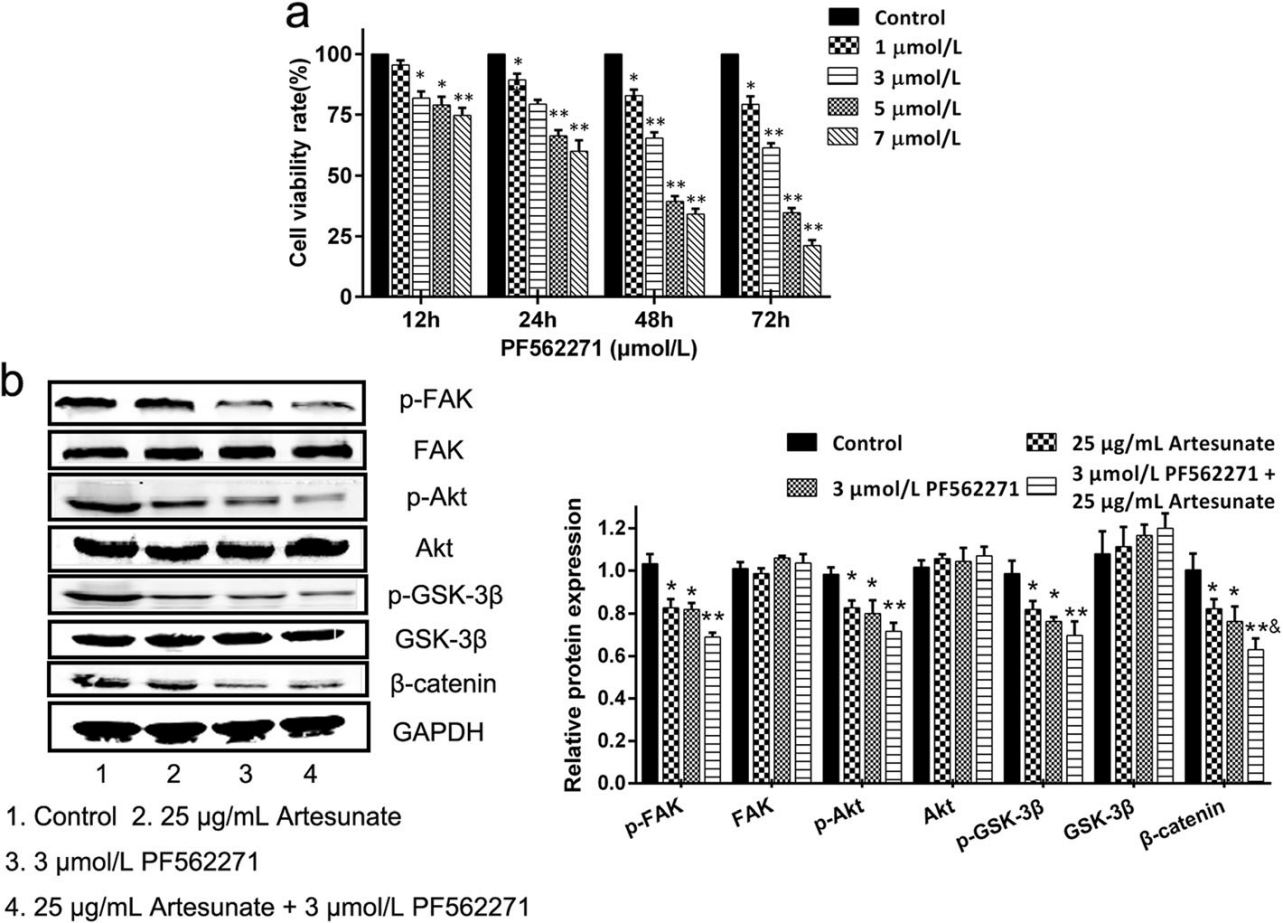
ART inhibits liver fibrosis via the FAK/Akt/β-catenin pathway. **a** The relative viability of LX-2 cells was determined using the CCK-8 assay after treatment with different concentrations of the FAK inhibitor PF562271 (0, 1, 3, 5 and 7 μmol/l) for 12, 24, 48 or 72 h (*n* = 6); **b** The cells were treated with 3 μM PF562271 alone, 25 μg/ml ART alone, or the combination of 25 μg/ml ART and 3 μM PF562271 for 24 h, then harvested. The levels of FAK, p-FAK, Akt, p-Akt, GSK-3β, p-GSK-3β and β-catenin were measured using western blotting (*n* = 4). Each column shows the mean ± SEM. ^*^ means *P* < 0.05 vs control. ^**^ means *P* < 0.01 vs control. ^&^ means *P* < 0.05 vs ART group

To verify that the FAK/Akt/β-catenin pathway underlies the observed anti-fibrogenic effects of ART, LX-2 cells were treated with the FAK inhibitor PF562271. First, we determined an appropriate dose of PF562271 for the following experiments. LX-2 cells were treated with various PF562271 concentrations (0, 1, 3, 5 and 7 μmol/l) for 12, 24, 48 or 72 h. Then, cell viability was measured using the CCK-8 assay. PF562271 markedly inhibited the growth of LX-2 cells in a concentration- and time-dependent manner (Fig. 5a). The IC_50_ of PF562271 was 12.68 μmol/l at 12 h, 8.64 μmol/l at 24 h, 4.70 μmol/l at 48 h and 3.45 μmol/l at 72 h. In order to ensure cell viability, 3 μmol/l PF562271 was used in subsequent experiments based on these results. LX-2 cultures were treated with 3 μmol/l PF562271 alone, 25 μg/ml ART alone, or the combination of 25 μg/ml ART and 3 μmol/l PF562271 for 24 h. Western blotting revealed that PF562271 led to reduced levels of phosphorylated FAK and inhibited signaling via the Akt/β-catenin pathway (Fig. 5b). Taken together, our analyses suggest that the FAK gene acts upstream of the Akt/β-catenin pathway, and that ART inhibits FAK phosphorylation, which in turn inhibits the expression of Akt/β-catenin, ultimately resulting in the anti-fibrogenic effects on LX-2 cells.

## Discussion

An increasing number of studies have explored the effects of Chinese medicine on hepatic fibrosis [29, 30], but only few studies to date have examined the anti-fibrogenic properties of ART. We herein demonstrated that ART inhibits the proliferation and activation of HSCs, promotes HSCs apoptosis, and provided evidence that ART exerts these effects by inhibiting the FAK/Akt/β-catenin pathway.

Inhibiting the proliferation of HSCs may be an effective strategy for arresting the progression of liver fibrosis at its source [31]. Our results suggest that ART strongly inhibits the proliferation of LX-2 cells in a dose- and time-dependent manner, consistent with previous findings [22]. Promoting the apoptosis of HSCs may be an effective method for inhibiting the progression of hepatic fibrosis. Our flow cytometry and western blotting experiments demonstrated that ART markedly increased the proportion of LX-2 cells in early apoptosis, and upregulated the Bax/Bcl-2 ratio. HSCs activation is characterized by excessive deposition of ECM proteins, including α-SMA and collagen I [32], and ART markedly attenuated the mRNA expression of the genes encoding α-SMA and collagen I in the present study. Our results suggest that ART exerts anti-proliferative, anti-activation and pro-apoptotic effects on HSCs.

We conducted molecular analyses to identify key molecules and pathways underlying these therapeutic effects of ART. First, we found evidence linking the anti-fibrogenic activity of ART to a reduction in the levels of p-FAK. In addition, our assays suggest that ART blocks the Akt/β-catenin pathway by downregulating the levels of p-Akt, p-GSK-3β and β-catenin in a dose-dependent manner, without affecting the levels of GSK-3β, which is consistent with previous findings [27]. The results demonstrated Artesunate, at least in part, was able to inhibit the phosphorylation levels of FAK. The FAK inhibitor PF562271, similar to ART, markedly decreased the levels of p-Akt, p-GSK-3β and β-catenin. These results support the hypothesis that the FAK gene acts upstream of the Akt/β-catenin pathway. Our experiments point to a mechanism in which ART inhibits the progression of liver fibrosis by inhibiting the FAK/Akt/β-catenin pathway.

In summary, our study demonstrated that ART can inhibit the proliferation and activation, as well as promote the apoptosis of the human HSC cell line LX-2, providing testable hypotheses on how this traditional medicinal agent ameliorates hepatic fibrosis. ART appears to exert its anti-fibrogenic effects, at least in part, by reducing the phosphorylation levels of FAK, Akt and GSK-3β. These results implicate the FAK/Akt/β-catenin signaling pathway in liver fibrosis.

There were certain limitations to the present study. First, there is yet no relevant study on the anti-fibrosis effects of ART in vivo. Second, it could not be fully demonstrated that the effects of ART on LX-2 was dependent on the FAK signaling pathway, as FAK was not activated or overexpressed. Third, it has not yet been investigated whether the TGF-β/Smad pathway, the classical pathway implicated in liver fibrosis, is involved in the anti-fibrogenic mechanism of ART. Therefore, future studies should be focused on verifying and extending the anti-fibrogenic effects of ART in vivo, as well as exploring other molecules and pathways through overexpressing or silencing of target genes that may contribute to fibrosis.

## Conclusions

In conclusion, ART dispalys anti-fibrosis effects in LX-2 cells by blocking FAK/Akt/β-catenin signaling pathway. We suggest that our present research provides novel insight into the treatment of hepatic fibrosis.

## Abbreviations

Akt: Protein kinase B
Art: Artesunate
CCK-8: Cell Counting Kit-8
DMSO: Dimethyl sulfoxide
ECM: Extracellular matrix
FAK: Focal adhesion kinase
HSCs: Hepatic stellate cells
PBS: Phosphate-buffered saline
RT-qPCR: Reverse transcription-quantitative polymerase chain reaction
SEM: Standard error of mean
TBST: Tris-buffered saline containing Tween-20
α-SMA: α-smooth muscle actin

## References

[1] Bataller R, Brenner DA (2005). Liver fibrosis. J Clin Investig.

[2] Arthur MJ (2002). Reversibility of liver fibrosis and cirrhosis following treatment for hepatitis C. Gastroenterology.

[3] Pellicoro A, Ramachandran P, Iredale JP (2012). Reversibility of liver fibrosis. Fibrogenesis Tissue Repair.

[4] Chen MF, Huang SJ, Huang CC, Liu PS, Lin KI, Liu CW, Hsieh WC, Shiu LY, Chen CH (2016). Saikosaponin d induces cell death through caspase-3-dependent, caspase-3-independent and mitochondrial pathways in mammalian hepatic stellate cells. BMC Cancer.

[5] Lv Z, Xu L (2011). Salvianolic Acid B Inhibits ERK and p38 MAPK Signaling in TGF-β1-Stimulated Human Hepatic Stellate Cell Line (LX-2) via Distinct Pathways. Evid Based Complement Alternat Med.

[6] Iredale JP, Benyon RC, Pickering J, McCullen M, Northrop M, Pawley S, Hovell C, Arthur MJ (1998). Mechanisms of spontaneous resolution of rat liver fibrosis. Hepatic stellate cell apoptosis and reduced hepatic expression of metalloproteinase inhibitors. J Clin Investig.

[7] Jiang HQ, Zhang XL, Liu L, Yang CC (2004). Relationship between focal adhesion kinase and hepatic stellate cell proliferation during rat hepatic fibrogenesis. World J Gastroenterol.

[8] Reif S, Lang A, Lindquist JN, Yata Y, Gabele E, Scanga A, Brenner DA, Rippe RA (2003). The role of focal adhesion kinase-phosphatidylinositol 3-kinase-Akt signaling in hepatic stellate cell proliferation and type I collagen expression. J Biol Chem.

[9] Parsons CJ, Takashima M, Rippe RA (2007). Molecular mechanisms of hepatic fibrogenesis. J Gastroenterol Hepato.

[10] Zhao XK, Yu L, Cheng ML, Che P, Lu YY, Zhang Q, Mu M, Li H, Zhu LL, Zhu JJ, Hu M, Li P, Liang YD, Luo XH, Cheng YJ, Xu ZX, Ding Q (2017). Focal adhesion kinase regulates hepatic stellate cell activation and liver fibrosis. Sci Rep.

[11] Liu XJ, Yang L, Wu HB, Qiang O, Huang MH, Wang YP (2002). Apoptosis of rat hepatic stellate cells induced by anti-focal adhesion kinase antibody. World J Gastroenterol.

[12] Liu L, Wei J, Huo XX, Fang SM, Yao DM, Gao JP, Jiang HQ, Zhang XL (2012). The Salvia miltiorrhiza monomer IH764-3 induces apoptosis of hepatic stellate cells in vivo in a bile duct ligation-induced model of liver fibrosis. Mol Med Rep.

[13] Zhang K, Jiang MN, Zhang CH, Li C, Jia YJ (2014). Effects of Ganfukang on expression of connective tissue growth factor and focal adhesion kinase/protein kinase B signal pathway in hepatic fibrosis rats. Chin J Integr Med.

[14] Dong D, Yin L, Qi Y, Xu L, Peng J (2015). Protective effect of the Total Saponins from Rosa laevigata Michx fruit against carbon tetrachloride-induced liver fibrosis in rats. Nutrients.

[15] Chen JS, Li HS, Huang JQ, Dong SH, Huang ZJ, Yi W, Zhan GF, Feng JT, Sun JC, Huang XH (2016). MicroRNA-379-5p inhibits tumor invasion and metastasis by targeting FAK/AKT signaling in hepatocellular carcinoma. Cancer Lett.

[16] Monga SP (2015). β-Catenin signaling and roles in liver homeostasis, injury, and tumorigenesis. Gastroenterology.

[17] Ge WS, Wang YJ, Wu JX, Fan JG, Chen YW, Zhu L (2014). β-Catenin is overexpressed in hepatic fibrosis and blockage of Wnt/β-catenin signaling inhibits hepatic stellate cell activation. Mol Med Rep.

[18] Li LN, Zhang HD, Yuan SJ, Tian ZY, Wang L, Sun ZX (2007). ARTsunate attenuates the growth of human colorectal carcinoma and inhibits hyperactive Wnt/beta-catenin pathway. Int J Cancer.

[19] Li W, Zhu C, Li Y, Wu Q, Gao R (2014). Mest attenuates CCl4-induced liver fibrosis in rats by inhibiting the Wnt/β-catenin signaling pathway. Gut Liver.

[20] MadanKumar P, NaveenKumar P, Manikandan S, Devaraj H, NiranjaliDevaraj S (2014). Morin ameliorates chemically induced liver fibrosis in vivo and inhibits stellate cell proliferation in vitro by suppressing Wnt/β-catenin signaling. Toxicol Appl Pharmacol.

[21] Cui L, Jia X, Zhou Q, Zhai X, Zhou Y, Zhu H (2014). Curcumin affects β-catenin pathway in hepatic stellate cell in vitro and in vivo. J Pharm Pharmacol.

[22] Xu Y, Liu W, Fang B, Gao S, Yan J (2014). Artesunate ameliorates hepatic fibrosis induced by bovine serum albumin in rats through regulating matrix metalloproteinases. Eur J Pharmacol.

[23] Lai L, Chen Y, Tian X, Li X, Zhang X, Lei J, Bi Y, Fang B, Song X (2015). Artesunate alleviates hepatic fibrosis induced by multiple pathogenic factors and inflammation through the inhibition of LPS/TLR4/NF-κB signaling pathway in rats. Eur J Pharmacol.

[24] Li HX, Liu H, Wang CM, Wang HJ, Chen J (2014). Artesunate restraining MAPK passage by smad7 to resist pulmonary fibrosis. Eur Rev Med Pharmacol Sci.

[25] Xu N, Zhou X, Wang S, Xu LL, Zhou HS, Liu XL (2015). Artesunate induces SKM-1 cells apoptosis by inhibiting hyperactive β-catenin signaling pathway. Int J Med Sci.

[26] Zhang H, Qi Q, Xiong YG, Zhang Y, Peng R (2015). Effects of Artesunate on expression of correlation factor of Wnt/β-catenin signaling pathway in rat hepatic stellate cells. Chin J Hosp Pharm.

[27] Bai RD, Zhang H, Huang CY (2017). Effect of Artesunate on Akt/GSK-3β/β-catenin signal pathway in human hepatic stellate cells. China Pharm.

[28] Kamo N, Ke B, Busuttil RW, Kupiec-Weglinski JW (2013). PTEN-mediated Akt/β-catenin/Foxo1 signaling regulates innate immune responses in mouse liver ischemia/reperfusion injury. Hepatology.

[29] Lee PJ, Woo SJ, Jee JG, Sung SH, Kim HP (2015). Bisdemethoxycurcumin induces apoptosis in activated hepatic stellate cells via cannabinoid receptor 2. Molecules.

[30] Zhang X, Xu Y, Chen JM, Liu C, Du GL, Zhang H, Chen GF, Jiang SL, Liu CH, Mu YP, Liu P (2017). Huang qi decoction prevents BDL-induced liver fibrosis through inhibition of notch signaling activation. Am J Chin Med.

[31] Chen MF, Huang CC, Liu PS, Chen CH, Shiu LY (2013). Saikosaponin a and saikosaponin d inhibit proliferation and migratory activity of rat HSC-T6 cells. J Med Food.

[32] Inagaki Y, Kushida M, Higashi K, Itoh J, Higashiyama R, Hong YY, Kawada N, Namikawa K, Kiyama H, Bou-Gharios G, Watanabe T, Okazaki I, Ikeda K (2005). Cell type-specific intervention of transforming growth factor beta/Smad signaling suppresses collagen gene expression and hepatic fibrosis in mice. Gastroenterology.

